# Vimentin Modulates Infectious Internalization of Human Papillomavirus 16 Pseudovirions

**DOI:** 10.1128/JVI.00307-17

**Published:** 2017-07-27

**Authors:** Georgia Schäfer, Lisa M. Graham, Dirk M. Lang, Melissa J. Blumenthal, Martina Bergant Marušič, Arieh A. Katz

**Affiliations:** a Division of Medical Biochemistry and Structural Biology, Department of Integrative Biomedical Sciences, University of Cape Town, Cape Town, South Africa; b Institute of Infectious Disease and Molecular Medicine, University of Cape Town, Cape Town, South Africa; c UCT Receptor Biology Research Unit, University of Cape Town, Cape Town, South Africa; d SA-MRC Gynecology Cancer Research Centre, Faculty of Health Sciences, University of Cape Town, Cape Town, South Africa; e Division of Physiological Sciences, Department of Human Biology, University of Cape Town, Cape Town, South Africa; f Laboratory for Environmental and Life Sciences, University of Nova Gorica, Nova Gorica, Slovenia; University of Illinois at Chicago

**Keywords:** HPV16, vimentin, pseudovirions, infectious internalization

## Abstract

Human papillomavirus (HPV) infection is the most common viral infection of the reproductive tract, with virtually all cases of cervical cancer being attributable to infection by oncogenic HPVs. However, the exact mechanism and receptors used by HPV to infect epithelial cells are controversial. The current entry model suggests that HPV initially attaches to heparan sulfate proteoglycans (HSPGs) at the cell surface, followed by conformational changes, cleavage by furin convertase, and subsequent transfer of the virus to an as-yet-unidentified high-affinity receptor. In line with this model, we established an *in vitro* infection system using the HSPG-deficient cell line pgsD677 together with HPV16 pseudovirions (HPV16-PsVs). While pgsD677 cells were nonpermissive for untreated HPV16-PsVs, furin cleavage of the particles led to a substantial increase in infection. Biochemical pulldown assays followed by mass spectrometry analysis showed that furin-precleaved HPV16-PsVs specifically interacted with surface-expressed vimentin on pgsD677 cells. We further demonstrated that both furin-precleaved and uncleaved HPV16-PsVs colocalized with surface-expressed vimentin on pgsD677, HeLa, HaCaT, and NIKS cells, while binding of incoming viral particles to soluble vimentin protein before infection led to a substantial decrease in viral uptake. Interestingly, decreasing cell surface vimentin by small interfering RNA (siRNA) knockdown in HeLa and NIKS cells significantly increased HPV16-PsV infectious internalization, while overexpression of vimentin had the opposite effect. The identification of vimentin as an HPV restriction factor enhances our understanding of the initial steps of HPV-host interaction and may lay the basis for the design of novel antiviral drugs preventing HPV internalization into epithelial cells.

**IMPORTANCE** Despite HPV being a highly prevalent sexually transmitted virus causing significant disease burden worldwide, particularly cancer of the cervix, cell surface events preceding oncogenic HPV internalization are poorly understood. We herein describe the identification of surface-expressed vimentin as a novel molecule not previously implicated in the infectious internalization of HPV16. Contrary to our expectations, vimentin was found to act not as a receptor but rather as a restriction factor dampening the initial steps of HPV16 infection. These results importantly contribute to our current understanding of the molecular events during the infectious internalization of HPV16 and open a new direction in the development of alternative drugs to prevent HPV infection.

## INTRODUCTION

High-risk human papillomaviruses (HPV) belong to the seven groups of viruses known to date which have been recognized to be consistently associated with various types of human cancer (1). HPV type 16 (HPV16), the most common oncogenic genotype, is an obligatory intracellular virus which has been extensively studied with regard to its entry mechanisms. While multiple possible receptors have been described, the exact infectious uptake of HPV16 into epithelial cells remains controversial (2, 3). The current 2-step entry model highlights the crucial role of heparan sulfate proteoglycans (HSPGs) as the initial attachment receptor situated within the plasma membrane or the extracellular matrix via engagement of the HPV major capsid protein L1 (4–10). In addition, laminin 332 secreted onto the extracellular matrix by keratinocytes has been proposed as a high-affinity initial attachment molecule (11). Binding to HSPGs induces conformational changes in the icosahedral capsid that facilitate proteolytic cleavage of L1 by the secreted serine protease kallikrein 8 (KLK8) (12). Subsequent interactions of the viral particle with the host cell chaperone cyclophilin B lead to the exposure of the N-terminal portion of the minor capsid protein L2 which contains a conserved consensus cleavage site for the host-encoded extracellular proprotein convertase furin (13). Cleavage of L2 by furin was found to be an indispensable step for HPV infection (14, 15). Although it is now accepted that furin cleavage of L2 plays a critical role for endosomal escape at later stages during the infection process rather than being a prerequisite at the early stages of binding and entry, it nevertheless leads to exposure of a cell surface receptor binding site on L1 (16, 17). This, together with the described changes in virion conformation, is postulated to reduce their affinity for HSPGs, thereby facilitating their transfer to and engagement with a secondary non-HSPG entry receptor(s), culminating in virions that are primed for infectious internalization (9, 18, 19). Interestingly, furin-precleaved viral particles were found to bind directly to the secondary receptor(s), thereby bypassing the necessity of HSPG interaction (20). Several secondary, predominantly L1-specific receptors have been proposed that may mediate infectious uptake, such as α6 integrin (21), epidermal growth factor receptor (EGFR), keratinocyte growth factor receptor (KGFR) (22), and tetraspanins (23). Moreover, with the identification of the annexin A2 heterotetramer (AIIt), a multifunctional protein involved in diverse cellular processes (24), an L2-specific receptor has been suggested (25) to regulate entry and intracellular trafficking of the virus (26). However, the precise cellular components mediating HPV uptake into permissive cells remain unknown.

While viral attachment to the host cell is rather quick, internalization of HPV was found to be an unusually slow and asynchronous process (27). Despite contradicting observations due to different experimental systems used to study virus entry, it is assumed that the endocytic uptake of HPV is dependent on actin dynamics but is clathrin, caveolin, cholesterol, and dynamin independent, implying a noncanonical internalization pathway related to macropinocytosis (27, 28). After internalization, the virus traffics through the endosomal system, interacting with sorting nexin 17 in the endosome (29, 30) as well as with retromer components (31) and γ-secretase in the *trans*-Golgi network (32, 33). In order to initiate infection, the viral genome complexed with L2 needs to escape from the endosomal compartment into the cytoplasm and travel to the nucleus via dynein-mediated transport along microtubules (34, 35). Effective translocation from the endosomes into the cytosol is dependent upon prior furin cleavage of L2 at the cell surface (14, 36, 37), demonstrating again the critical role of furin cleavage in HPV entry.

In an attempt to identify novel host molecules that interact with furin-precleaved (FPC) HPV16 particles at the stage of cell entry, we performed biochemical pulldown experiments and found vimentin expressed at the host cell surface to be a potential candidate modulating viral uptake. Vimentin is a type III intermediate filament (IF) protein and the major IF of mesenchymal cells and cells adapted to tissue culture as well as transformed cell lines (38). Unlike microfilaments and microtubules, IFs are a chemically heterogeneous group of structural proteins (39). Besides its traditionally assigned functions in maintaining the mechanical and structural properties of cells, vimentin also acts as a key organizer in the context of intracellular dynamics and architecture, such as cell adhesion, cell migration, and cell signaling (40). Interestingly, several viruses exploit vimentin during different phases of their life cycle, ranging from viral entry to replication, virus assembly, and egress (39). As one of the major components of the cytoskeleton, vimentin is primarily located in the cytoplasm, especially around the plasma membrane. However, vimentin has also been found to be expressed on the cell surface and in the extracellular space in several cell types (40). While the biological functions of surface-expressed vimentin still remain to be elucidated, an increasing number of reports suggest a conserved role for cell surface vimentin as a component of the attachment and uptake complex of several viruses (41–48). Also, bacteria interact with surface vimentin to mediate cellular attachment (49), for example, Escherichia coli and group A streptococci (50, 51), while Mycobacterium tuberculosis-infected monocytes are recognized by natural killer cells through surface-expressed vimentin (52).

Here we report the identification of surface-expressed vimentin for binding of both furin-precleaved and uncleaved HPV16-PsVs modulating infectious internalization of the incoming virions.

## RESULTS

### Identification of cell surface vimentin as a binding protein for HPV16-PsVs.

**FIG 1:**
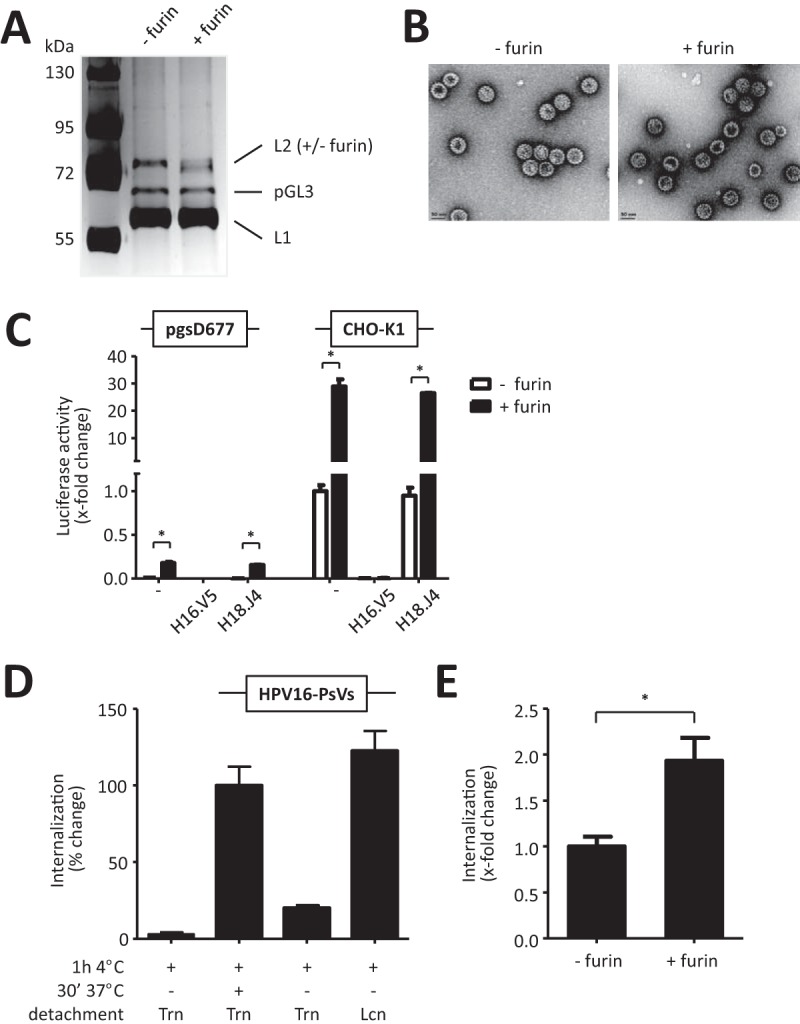
Establishing the experimental conditions for subsequent identification of HPV16-PsV-binding surface molecules. HPV16 pseudovirions encapsidating the luciferase reporter gene plasmid pGL3-control were treated with furin or left untreated. (A) Purity and successful furin treatment of the PsV preparations (1.5 μg per lane) was assessed by SDS-PAGE and subsequent silver staining. Note that the additional band at approximately 60 kDa is the silver-stained pGL3-control plasmid (70). The leftmost lane shows molecular mass markers, in kilodaltons. (B) Negative EM staining of furin-treated and untreated HPV16-PsVs. (C) HSPG-deficient pgsD677 cells and their corresponding wild-type CHO-K1 cells were infected with furin-treated or untreated HPV16-PsVs. In control experiments, the virions were preincubated with either of the neutralizing antibodies H16.V5 and H18.J4. Luciferase activity of the cell lysates was measured 48 h postinfection and normalized against total protein concentration and is presented as x-fold change relative to control infection of CHO-K1 cells with untreated HPV16-PsVs, which was set as 1. (D) Quantification of viral internalization was performed by flow cytometry of pgsD677 cells infected with Alexa Fluor 488 (AF488)-conjugated HPV16-PsVs for 1 h on ice followed by 30 min at 37°C. Cells were lifted with trypsin-EDTA (Trn) to remove surface-bound virions. In control experiments, cells were either not shifted to 37°C to avoid internalization or lifted with lidocaine hydrochloride-EDTA (Lcn), which does not remove surface-bound particles. Experiments were performed in triplicate, and results were quantified by quadrant analysis of the dot plot of three independent experiments and are presented as percent change relative to the mean fluorescence intensity of cells lifted with Trn, which was set as 100%. (E) Internalization of furin-pretreated or untreated AF488-conjugated HPV16-PsVs by pgsD677 cells as determined by flow cytometry as described for panel D. Significance was calculated by means of the two-tailed Student *t* test from three independent experiments performed in triplicate, and a *P* value of ≤0.05 (*) was regarded as statistically significant.

While the extracellular events preceding HPV uptake into host cells are relatively well understood (4–13, 18–20), the nature of the specific entry receptor is still widely debated. In an attempt to biochemically pull down cell surface molecules that interact with and potentially mediate uptake of the virus, we established an experimental system that bypasses the initial extracellular attachment steps necessary for conformational changes that ultimately lead to furin cleavage of L2 and viral uptake. We generated furin-precleaved HPV16 pseudovirions which expectedly contain some (although not all [17]) L2 proteins with proteolytically processed N termini (Fig. 1A). Importantly, no copurifications were visible by SDS-PAGE with silver staining, thereby minimizing the possibility of furin targeting other proteins besides the virus capsids (Fig. 1A).

Although we could not detect any obvious morphological differences between uncleaved and FPC HPV16-PsVs by negative electron microscopic (EM) staining (Fig. 1B), furin cleavage had a substantial functional impact on infection of the HSPG-deficient cell line pgsD677: while pgsD677 cells were practically noninfectible by HPV16-PsVs, furin cleavage of the particles led to an approximately 40-fold increase in infection as measured by luciferase reporter gene activity (Fig. 1C). Moreover, infection of CHO-K1 wild-type cells also resulted in a more robust (approximately 30-fold) increase of infection in the presence of FPC particles, while neutralization with the HPV16-neutralizing antibody H16.V5 (but not with the HPV18-neutralizing antibody H18.J4) abolished infectious uptake independently of furin pretreatment as expected (53) in both cell types (Fig. 1C). These experiments not only demonstrated the impact of furin treatment on HPV16-PsV infectivity but also confirmed the suitability of pgsD677 cells together with FPC HPV16-PsVs as an HSPG-independent infection system (17).

In order to study early steps in HPV infection involving quantification of virus internalization, we tested the effect of trypsin-EDTA on the removal of surface-bound but not internalized particles. When analyzed by flow cytometry, binding of Alexa Fluor 488 succinimidyl ester (AF488)-labeled HPV16-PsVs to pgsD677 cells for 1 h at 4°C was found to be almost completely removed by treatment with trypsin-EDTA but not with lidocaine hydrochloride-EDTA (Fig. 1D). However, internalization of the particles was well detected when cells were subsequently shifted to 37°C for 30 min and treated with trypsin-EDTA, almost reaching the levels seen when cells were only allowed to bind for 1 h at 4°C and lifted with lidocaine hydrochloride-EDTA (Fig. 1D). These results were also confirmed with all other cell lines used in this study (data not shown) and demonstrated the suitability of trypsin digestion for removal of surface-bound HPV16-PsVs, allowing the quantification of their internalization. Interestingly, furin pretreatment of the viral particles not only substantially affected infectivity of pgsD677 cells (Fig. 1C) but also increased FPC HPV16-PsV internalization as measured by flow cytometry using AF488-labeled virions (Fig. 1E). These data confirmed that FPC HPV16-PsVs can bypass the requirement for HSPG engagement during infectious uptake, thereby permitting direct binding to the still elusive secondary receptor (17).

**FIG 2:**
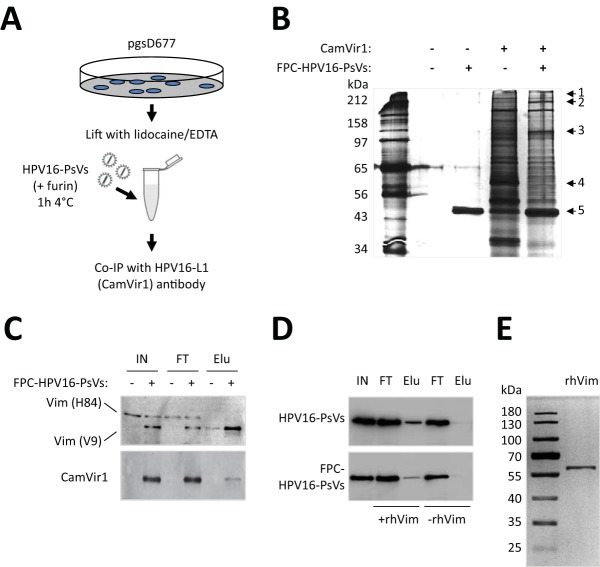
Identification of cell surface vimentin as a binding protein for HPV16-PsVs. (A) Schematic outline of the pulldown experiment to isolate FPC HPV16-PsVs-binding surface proteins. (B) Silver-stained SDS-PAGE gel of pgsD677 surface proteins bound to FPC HPV16-PsVs pulled down by the HPV16-L1-specific antibody CamVir1. Numbers on the right side refer to protein bands specific to FPC HPV16-PsV interaction, which were determined visually by comparing to control pgsD677 cells that were not incubated with HPV16-PsVs before immunoprecipitation. Candidate bands were excised from the gel and identified by MALDI-TOF mass spectrometry analysis: 1, tetratricopeptide repeat protein 39B OS (Rattus norvegicus) (protein significance score: 42); 2, guanine nucleotide-binding protein G(I)/G(S)/G(O) subunit gamma-5-like (Mus musculus) (protein significance score: 36); 3, immunoglobulin heavy chain (Mus musculus) (protein significance score: 40); 4, vimentin (Rattus norvegicus) (protein significance score: 139); 5, γ-actin (Mus musculus) (protein significance score: 88). The leftmost lane shows molecular mass markers, in kilodaltons. (C) Western blot of pulldown experiment (as in panel B), probed simultaneously with the antivimentin antibodies H84 and V9 (top) and reprobed with the antibody CamVir1 (bottom). Note that in the presence of HPV16-PsVs the size of the vimentin protein is slightly reduced. IN, input; FT, flowthrough; Elu, eluate. (D) Pulldown of HPV16-PsVs (with or without furin pretreatment) using immobilized recombinant human vimentin protein (rhVim). Proteins were detected by Western blotting using the antibody CamVir1. (E) Silver-stained SDS-PAGE gel of 0.5 μg of recombinant human vimentin protein. The leftmost lane shows molecular mass markers in kilodaltons.

We therefore performed immunoprecipitation (IP) assays of live pgsD677 cells incubated with FPC HPV16-PsVs using the HPV16-L1-specific antibody CamVir1 (Fig. 2A). Precipitated proteins were separated by SDS-PAGE followed by silver staining of the gel, allowing visual comparison to appropriate controls (Fig. 2B). Candidate protein bands were excised, processed for matrix-assisted laser desorption ionization–time of flight mass spectrometry (MALDI-TOF) analysis, and identified using the Matrix Science Database (MSDB) and searching the NCBI database. Among the molecules identified, vimentin received the highest protein significance score, 139, and was considered an attractive candidate involved in HPV recognition and binding due to its involvement in the attachment and uptake of several other viruses and bacteria when expressed at the cell surface (41–48, 50–52). In order to validate the involvement of pgsD677 cell surface vimentin in FPC HPV16-PsV binding, Western blots based on the immunoprecipitation experiment shown in Fig. 2B were performed. Interestingly, when probing with antibodies raised against different vimentin epitopes (i.e., H84 versus V9), we found that the presence of the viral particles led to a slight reduction in the size of the precipitated vimentin protein (Fig. 2C). No such protein was precipitated in the absence of FPC HPV16-PsVs. Unspecific binding of the vimentin V9 antibody to the HPV16-L1 protein (both being of similar size) was excluded in appropriate control experiments (data not shown). As it is possible that the observed different vimentin fragments were artifacts caused by proteolysis during cell isolation (54, 55), we performed pulldown of the viral particles in a cell-free context in the presence or absence of immobilized recombinant human vimentin protein (Fig. 2D). The purity of the vimentin protein was confirmed by silver staining following SDS-PAGE (Fig. 2E), thereby excluding virus binding to other molecules besides vimentin. These experiments confirmed that HPV16-PsVs directly interacted with vimentin. Interestingly, this interaction was found to be independent of furin pretreatment of the viral particles.

### Surface vimentin colocalizes with HPV16-PsVs and affects viral uptake by pgsD677 cells.

**FIG 3:**
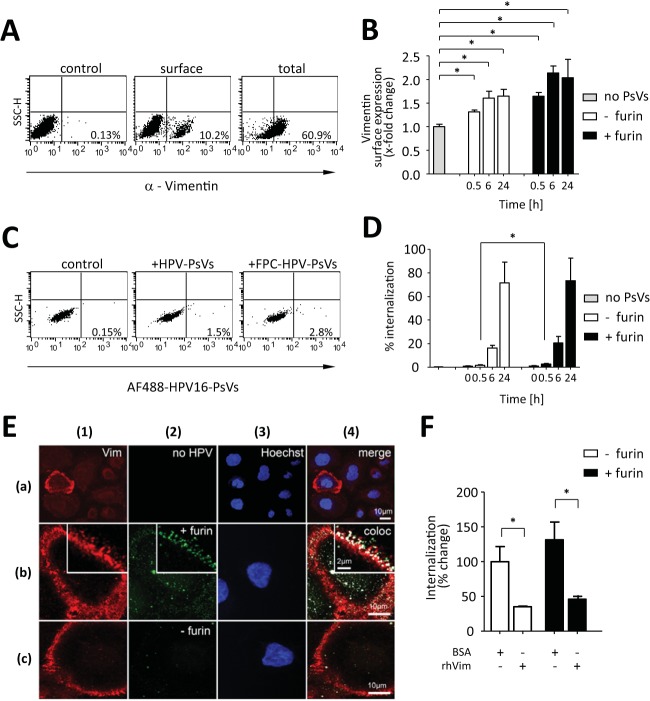
Surface vimentin modulates uptake of HPV16-PsVs by pgsD677 cells. AF488-conjugated HPV16-PsVs were used in all qualitative and quantitative assessments of infectious internalization by pgsD677 cells. Significance was calculated by means of the two-tailed Student *t* test from three independent experiments performed in triplicate; *P* values of ≤0.05 (*) were regarded as statistically significant. (A) Cell surface versus total vimentin expression was determined by flow cytometry and quantified using quadrant statistics of three independent experiments. Shown are representative dot plots with the average percentage of the mean fluorescence intensities of vimentin-positive cells in the lower right quadrant. (B) Quantification of vimentin surface expression after incubation of pgsD677 cells with HPV16-PsVs (with or without prior furin cleavage) for the indicated periods. (C) Flow cytometry analysis of pgsD677 cells with bound AF488-conjugated HPV16-PsVs (pretreated or not with furin). Quantification was done using quadrant statistics analysis. Shown are representative dot plots with the average percentage of the mean fluorescence intensities of AF488-positive cells in the lower right quadrant. (D) Quantification of internalization of HPV16-PsVs (with or without prior furin cleavage) for the indicated periods. (E) Confocal imaging of pgsD677 cells showing surface vimentin (column 1, red), which is present only in a subpopulation of cells (indicated by Hoechst nuclear stain, blue, column 3) (a) and colocalization analysis of cells stained for cell surface vimentin (column 1, red) and bound by AF488-conjugated HPV16-PsVs (with or without prior furin cleavage, column 2, green) for 1 h at 4°C (b and c). Nuclei were stained with Hoechst to indicate presence of cells. Signal overlays in column 4 indicate colocalization of the viral particles with cell surface vimentin as white pixels. High-magnification insets in columns 1, 2, and 4 show a detail of signal distribution and colocalization at the filopodial edge of the cell in a single optical section. Shown are representative images. (F) AF488-conjugated HPV16-PsVs (with or without prior furin cleavage) were pretreated with soluble recombinant human vimentin protein or BSA before infection of pgsD677 cells. Internalization of viral particles was assessed by flow cytometry and quantified using quadrant statistics and is presented as change in mean fluorescence intensity of AF488-positive cells compared to that of untreated particles, which was set as 100%.

To determine if vimentin could act as a surface molecule involved in HPV uptake, we initially characterized vimentin expression in pgsD677 cells and studied binding of AF488-labeled HPV16-PsVs (both furin treated and untreated) by flow cytometry and confocal microscopy. The expression pattern of surface vimentin agrees with the literature, as only a small percentage of cells presented detectable vimentin at the surface (Fig. 3A), which appeared polarized (punctate) at the cell membrane (44, 56), with a striking localization of vimentin to filopodia near the substrate (Fig. 3E, column 1). Due to the lack of HSPGs on pgsD677 cells, total binding of the viral particles to the cells was relatively low (Fig. 3C); however, individual cells expressing vimentin at the surface showed impressively high binding of the viral particles, with the majority displaying colocalization with vimentin (Fig. 3E, rows b and c). Generally, increased binding to pgsD677 cells was observed for FPC HPV16-PsVs compared to uncleaved particles (Fig. 3C and E, rows b and c), although levels of colocalization with vimentin were comparable: 85.3% (±20.3%) of FPC HPV16-PsVs and 78.7% (±35.1%) of uncleaved particles, respectively, colocalized with surface-expressed vimentin (Table 1). Interestingly, vimentin surface levels slightly increased over time upon infection with the viral particles; however, furin-precleaved and untreated particles had a comparable effects (Fig. 3B). Although initial (30 min) internalization of FPC HPV16-PsVs was increased compared to untreated particles (Fig. 1E), the two virus preparations were internalized to similar extents over a time course of 24 h (Fig. 3D).

**TABLE 1:** Quantification of HPV16-PsVs colocalization with surface vimentin on pgsD677, HaCaT, HeLa, and NIKS cells^*a*^

Cell line (viral prepn)	Colocalization coefficient for vimentin (SD)	Colocalization coefficient for virus (SD)	Overlap coefficient (SD)
pgsD677 (+HPV16-PsVs)	0.098 (0.044)	0.787 (0.351)	0.41 (0.32)
pgsD677 (+FPC HPV16-PsVs)	0.130 (0.060)	0.853 (0.203)	0.37 (0.13)
HaCaT (+HPV16-PsVs)	0.010 (0.008)	0.353 (0.165)	0.52 (0.36)
HaCaT (+FPC HPV16-PsVs)	0.014 (0.013)	0.319 (0.195)	0.43 (0.22)
HeLa (+HPV16-PsVs)	0.013 (0.011)	0.387 (0.099)	0.49 (0.19)
HeLa (+FPC HPV16-PsVs)	0.019 (0.012)	0.361 (0.105)	0.66 (0.23)
NIKS (+HPV16-PsVs)	0.011 (0.013)	0.808 (0.344)	0.46 (0.19)
NIKS (+FPC HPV16-PsVs)	0.018 (0.009)	0.707 (0.220)	0.51 (0.21)

a Binding of the indicated virus preparations (with or without furin treatment) to approximately 50 cells per cell line was analyzed by confocal microscopy in two independent experiments. Manders colocalization coefficients for vimentin (i.e., amount of vimentin colocalizing with virus) and virus (i.e., amount of virus colocalizing with vimentin) are presented, along with Manders overlap coefficients; a value of 0 indicates no colocalization, and a value of 1 indicates maximal colocalization.

In order to study any functional involvement of vimentin for infectious HPV internalization, we preincubated both untreated and FPC HPV16-PsVs with soluble human recombinant vimentin protein before infection. This led to a significant reduction in viral internalization as determined by flow cytometry (Fig. 3F). However, infectivity as measured by luciferase activity was not affected (data not shown), possibly due to dissociation of the vimentin-virus complexes by the time luciferase activity was measured (48 h postinfection).

### Vimentin modulates uptake of HPV16-PsVs in epithelial cells.

Although they were found suitable in this study as a cell system to investigate HPV infection at the immediate-uptake stage (by bypassing the initial attachment steps due to their lack of HSPGs), pgsD677 cells are nonhuman and nonkeratinocytes and thereby rather unrelated to the natural infectious environment for HPV. We therefore set out to confirm our initial observations using three cell lines representing physiologically relevant cell culture models: the human cervical cancer cell line HeLa and the human immortalized keratinocyte cell lines HaCaT and NIKS, two of the most widely accepted keratinocyte models for studying HPV infection (57). Unlike other immortalized cell lines, NIKS cells retain cell-type-specific growth requirements and differentiation properties, are nontumorigenic, and are virus free (58). Most importantly, NIKS cells were found to support the complete HPV life cycle (59).

**FIG 4:**
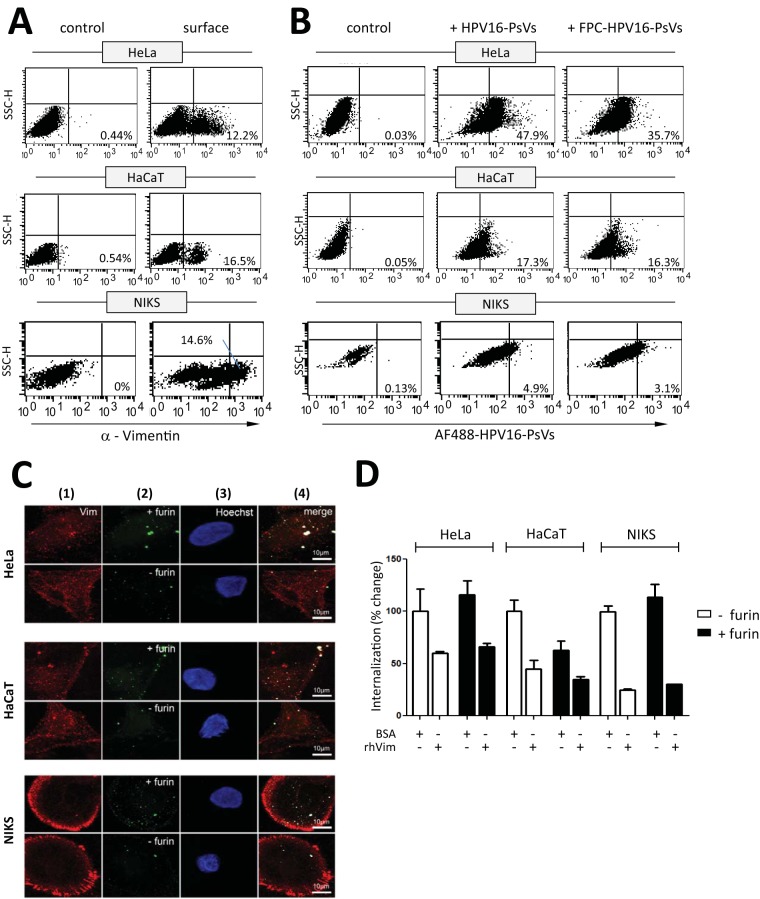
Vimentin expression and its role in HPV16-PsV infection of epithelial cells. (A) Cell surface vimentin expression of HeLa, HaCaT, and NIKS cells was determined by flow cytometry and quantified using quadrant statistics of three independent experiments. Shown are representative dot plots with the average percentage of the mean fluorescence intensities of vimentin-positive cells in the lower right quadrant. (B) Flow cytometry analysis of HeLa, HaCaT, and NIKS cells with bound AF488-conjugated HPV16-PsVs (with or without prior furin cleavage). Quantification was done using quadrant statistics analysis. Shown are representative dot plots with the average percentage of the mean fluorescence intensities of AF488-positive cells in the lower right quadrant. (C) Confocal imaging of HeLa, HaCaT, and NIKS cells stained for cell surface vimentin (column 1, red) and bound by AF488-conjugated HPV16-PsVs (with or without prior furin cleavage, column 2, green) for 1 h at 4°C. Nuclei were stained with Hoechst to indicate presence of cells (column 3, blue). Colocalization of the viral particles with cell surface vimentin is indicated by overlay of white pixels in the merged red and green channels (column 4). Shown are representative images. (D) AF488-conjugated HPV16-PsVs (with or without prior furin cleavage) were pretreated with soluble recombinant human vimentin protein or BSA before infection of HeLa, HaCaT, and NIKS cells. Internalization of viral particles was assessed by flow cytometry and is presented as percent change in mean fluorescence intensity of AF488-positive cells compared to that of untreated particles, which was set as 100%.

To verify cell surface expression of vimentin, we performed both flow cytometry and immunofluorescence microscopy on HeLa, HaCaT, and NIKS cells. Similar to the results for pgsD677 cells (Fig. 3A), only a small fraction of cells stained positive for surface vimentin (Fig. 4A), which appeared unevenly scattered over the cell surface, with some areas of high vimentin density (Fig. 4C). Unlike for pgsD677 cells (Fig. 3C), binding of HPV16-PsVs to HeLa, HaCaT, and NIKS cells was found to be much higher over the entire cell population as observed by flow cytometry, as attachment of the viral particles to HSPGs at the cell surface could occur (Fig. 4B). Interestingly, binding of untreated and FPC HPV16-PsVs occurred at comparable extents for the individual cell systems (Fig. 4B), with HeLa > HaCaT > NIKS. This cell line-specific trend was also reflected at the level of luciferase activity being a readout for infectivity (57) (data not shown), with furin treatment largely increasing infection in the individual cell system (Fig. 1C). However, individual vimentin-positive cells showed comparable particle-binding patterns of both untreated and FPC HPV16-PsVs when analyzed by confocal microscopy (Fig. 4C) in the three cell lines. Again, clear colocalization of AF488-labeled viral particles to surface-expressed vimentin was observed on HeLa, HaCaT, and NIKS cells (Fig. 4C), with no obvious difference between uncleaved and FPC HPV16-PsVs (Table 1).

Having demonstrated that all three cell lines expressed vimentin at the cell surface which colocalized with the viral particles (Fig. 4A and C), we next set out to investigate whether it also affected HPV16-PsV internalization, leading to infection. We preincubated the viral particles with soluble human recombinant vimentin protein before infection. As already seen for pgsD677 cells (Fig. 3F), this treatment led to a substantial reduction in viral internalization in all cell systems (Fig. 4D); however, infectivity was not affected for the reasons mentioned above (data not shown). Furin pretreatment did not influence virion internalization (Fig. 4D), unlike what was seen in pgsD677 cells (Fig. 1E), which is consistent with expression of HSPG in these human cells.

**FIG 5:**
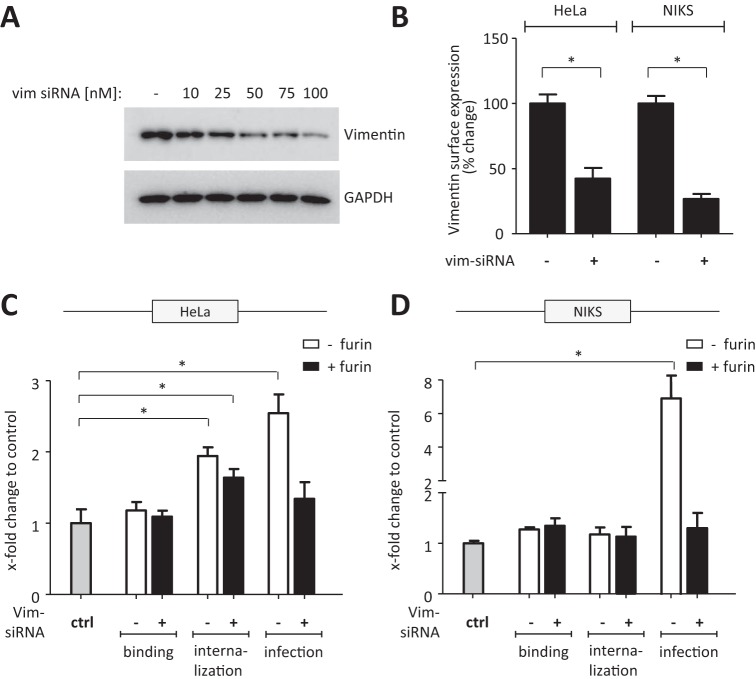
Decreased surface vimentin facilitates HPV16-PsV infectivity. HeLa and NIKS cells were reversely transfected with human vimentin siRNA for transient knockdown. (A) Western blot of HeLa cell lysates prepared 72 h after transfection with increasing concentrations of vimentin siRNA. Total vimentin was detected by the antivimentin antibody V9; glyceraldehyde-3-phosphate dehydrogenase (GAPDH) served as a loading control. (B) Cell surface expression of vimentin after knockdown with 100 nM siRNA for 72 h was determined by flow cytometry and quantified using quadrant statistics of three independent experiments and is presented as change in surface expression of the vimentin-positive cell population relative to cells transfected with scrambled (control) siRNA, which was set as 100%. (C and D) Quantification of viral binding and internalization was performed by flow cytometry of HeLa (C) and NIKS (D) cells transfected with 100 nM vimentin siRNA for 72 h and exposed to furin-pretreated or untreated AF488-conjugated HPV16-PsVs. Quadrant analysis of the dot plots of three independent experiments performed in triplicate is presented as x-fold change relative to the mean fluorescence intensity of cells transfected with control (scrambled) siRNA and infected with either furin-treated or untreated particles, as appropriate, which was set as 1 for all conditions. For quantification of infection, the cells were transfected with 100 nM vimentin siRNA for 72 h and infected with furin-pretreated or untreated HPV16-PsVs. Forty-eight hours postinfection, luciferase activity was measured and was normalized against total protein concentration and is presented as x-fold change relative to cells transfected with control (scrambled) siRNA and infected with either furin-treated or untreated particles, as appropriate, which was set as 1. Significance was calculated by means of the two-tailed Student *t* test, and a *P* value of ≤0.05 (*) was regarded as statistically significant.

**FIG 6:**
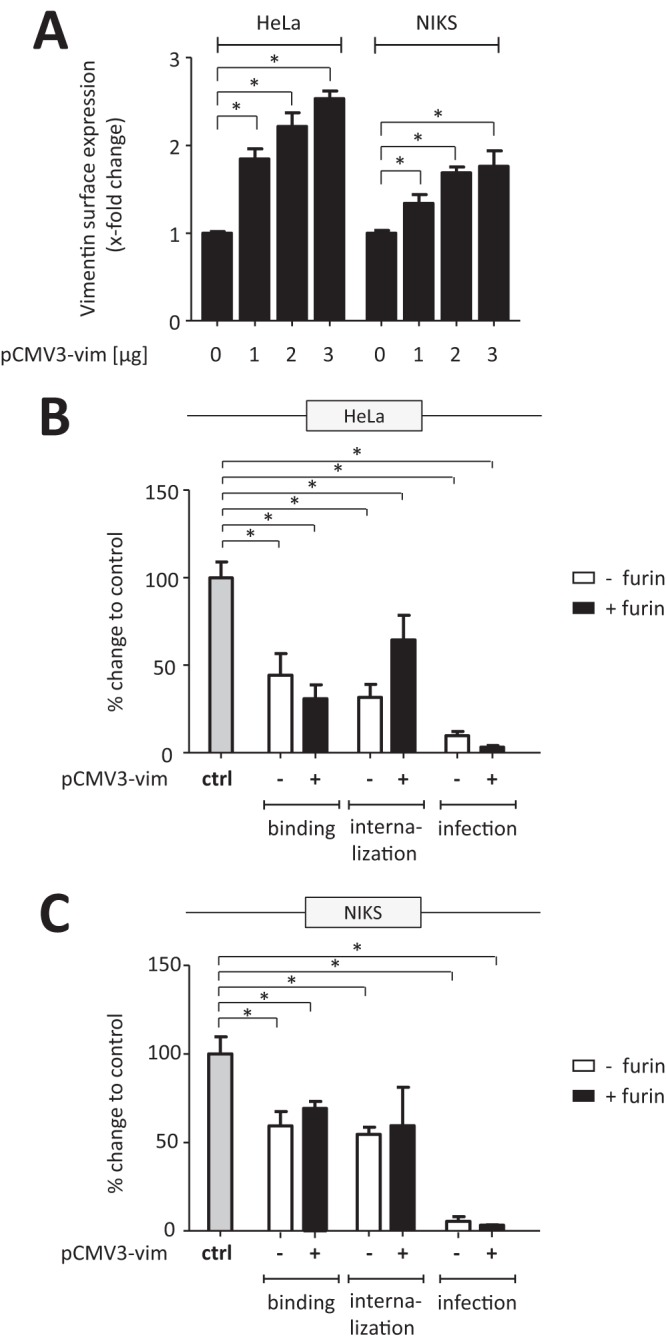
Surface vimentin acts as a viral restriction factor in HPV16-PsV infection. HeLa and NIKS cells were transiently transfected with plasmid pCMV3-vim for overexpression studies. (A) Cell surface expression of vimentin after transfection with increasing concentrations of plasmid pCMV3-vim for 14 h was determined by flow cytometry and quantified using quadrant statistics of three independent experiments and is presented as x-fold change in surface expression relative to control cells, which was set as 1. (B and C) Quantification of viral binding and internalization was performed by flow cytometry of HeLa (B) and NIKS (C) cells transfected with 1 μg of pCMV3-vim for 14 h and exposed to furin-pretreated or untreated AF488-conjugated HPV16-PsVs. Quadrant analysis of the dot plots of three independent experiments performed in triplicate is presented as percent change relative to the mean fluorescence intensity of control cells infected with either furin-treated or untreated particles, as appropriate, which was set as 100% for all conditions. For quantification of infection, the cells were transfected with 1 μg of pCMV3-vim for 14 h and infected with furin-pretreated or untreated HPV16-PsVs. Forty-eight hours postinfection, both floating and adherent cells were harvested to measure luciferase activity, which was normalized against total protein concentration and is presented as percent change relative to control cells infected with either furin-treated or untreated particles, as appropriate, which was set as 100%. Significance was calculated by means of the two-tailed Student *t* test, and a *P* value of ≤0.05 (*) was regarded as statistically significant.

The observed reduction in viral uptake upon preincubation of the virus particles with soluble vimentin (Fig. 3F and 4D) could be explained either by competition for surface-expressed vimentin (if vimentin functions as an uptake molecule), by a sequestering function vimentin employs toward infectious HPV particles, or by interference of vimentin with viral binding to the still-unknown entry receptor. We therefore altered vimentin expression in HeLa and NIKS cells by either small interfering RNA (siRNA) knockdown or overexpression. Interestingly, reduced vimentin expression upon knockdown by vimentin-specific siRNA (Fig. 5A and B) led to an increase in HPV16-PsV binding and internalization which was independent of prior furin treatment (Fig. 5C and D). Likewise, infection as measured by luciferase activity was significantly increased when vimentin expression was reduced, which was more pronounced when the particles were not pretreated with furin (Fig. 5C and D). Consistent with the observed increase in viral internalization upon vimentin knockdown, increasing the amount of surface vimentin by transient overexpression (Fig. 6A) significantly decreased HPV16-PsV binding and uptake independently of prior furin treatment (Fig. 6B and C). This was also accompanied by significant decreases in luciferase activity in both cell systems (Fig. 6B and C).

## DISCUSSION

Engagement with host cell surface molecules is one of the initial requirements for a successful viral infection; therefore, targeting viral receptors and identifying novel molecules that prevent receptor engagement by the incoming viral particles are promising strategies for the control of several viral infections. In the case of HPV, it has been assumed that the virions are unable to bind to their entry receptor until they have undergone an HSPG-dependent conformational change and subsequent furin cleavage (8). While HSPGs are expressed at the cell surface and are used by HPV as cellular attachment molecules *in vitro*, it is assumed that the conformational changes of the HPV capsid required for infection occur HSPG dependently on the basement membrane *in vivo* prior to the transit of the virions to the host cell surface (7). In order to bypass the need for these extracellular steps, we set up an experimental infection system using the HSPG-deficient cell line pgsD677 together with furin-precleaved HPV16 pseudovirions (FPC HPV16-PsVs). This model allowed us to biochemically pull down cellular surface molecules that the FPC particles directly interacted with, potentially including the still-elusive non-HSPG secondary entry receptor (20). One of the most promising candidate molecules that was precipitated together with FPC HPV16-PsVs was identified as vimentin, which, in addition to its function as a cytoskeletal filament protein, plays important roles in entry of, infection with, and/or replication of several viruses (39). Although vimentin is primarily expressed intracellularly, we and others observed surface expression on a subpopulation of cultured cells displaying a particular punctuate expression pattern (40). Importantly, several viral pathogens, including picornaviruses, mammalian porcine reproductive and respiratory syndrome virus (PRRSV), cowpea mosaic virus (CPMV), Japanese encephalitis virus (JEV), severe acute respiratory syndrome coronavirus (SARS-CoV), enterovirus 71, Theiler's virus, and human immunodeficiency virus 1 (HIV-1), use vimentin as a component of their cellular attachment and/or receptor machinery (41–46, 48, 60).

The physical interaction between FPC HPV16-PsVs and vimentin initially observed by immunoprecipitation was confirmed by immunofluorescence confocal microscopy as colocalization of the viral particles with surface-expressed vimentin on pgsD677 cells and on selected epithelial cell culture models, namely, HeLa, HaCaT, and NIKS, the last being particularly physiologically relevant for HPV infection, as NIKS cells retain cell-type-specific growth requirements and differentiation properties. Contrary to our expectation, however, the presence of vimentin was not found to aid in viral internalization but rather functioned as an inhibitory molecule. Both preincubation of the viral particles with soluble recombinant vimentin protein and overexpression of vimentin on the cell surface led to a substantial decrease in viral uptake and infection. In contrast, diminishing surface-expressed vimentin by siRNA knockdown significantly increased infectious internalization of the particles.

The functional significance of vimentin during natural HPV infection still needs to be elucidated, as cell culture systems do not fully represent the series of changes the virus undergoes *in vivo* after binding to the basement membrane prior to keratinocyte infection. We hypothesize that cell surface vimentin binding to viral particles plays a role in modulating the HPV entry process, possibly by interfering with the binding of the viral particle with the (still unknown) viral binding and uptake molecules. Although novel in the context of HPV infection, extracellular vimentin has been shown to be involved in the response to pathogens through interactions with incoming bacteria and soluble factors, thereby contributing to bacterial killing (54). Similar to the dual role of lectins in virus biology—lectins can be membrane associated or soluble and are known to be involved in either immune defense through neutralization and clearance of viral infection or can be exploited as entry molecules to facilitate viral spread (61)—vimentin may also exert dual effects on viral infection. While surface-expressed vimentin plays a role in the attachment and uptake machinery of several viruses as mentioned above (41–46, 48, 60), it appears to interfere with crucial steps in the HPV16 pseudovirus infection process at the level of binding and internalization into epithelial cells, possibly by either masking portions of the virus that interact with its receptor or by causing steric hindrance, thereby destabilizing or preventing receptor-virus interaction necessary for successful infection. Interestingly, vimentin is known to be associated with several integrins, such as α6 integrin (62), which has been proposed as a potential HPV receptor (21) and whose function in HPV uptake might be influenced by the presence of vimentin.

In this study, we focused on HPV16, the most common and best-characterized oncogenic HPV genotype. It is possible that some of the entry steps, including the role of vimentin, differ among HPV serotypes (2, 63). The participation of different receptors and/or modulating molecules can therefore account for differences in pathogenic profiles and differences in cell tropism. It is also interesting that HPV16 pseudovirus binding to recombinant vimentin protein as well as internalization by epithelial cells was little influenced by pretreatment of the viral particles with furin. While furin cleavage of L2 is indispensable for successful infection, as demonstrated on HSPG-deficient pgsD677 cells, the fact that untreated particles still enter the cells confirms the current notion that furin cleavage is important for endosomal escape rather than at the early stages of the infectious entry (14, 15). We therefore propose that the interaction between HPV16-PsVs and vimentin is independent of furin cleavage. Moreover, as the observed binding and internalization behavior of HPV16-PsVs did not greatly differ between the HSPG-expressing cell lines used in this study, we hypothesize that surface vimentin may act as a general viral restriction factor rather than a cell-type-specific one.

Knowledge of the early events in HPV infection, such as cellular surface molecule binding and its consequences for entry into susceptible cells, aids in the design of novel strategies for blocking HPV infection. Although the prophylactic vaccines that target certain HPV types show great efficacy (64), they are too costly for general use worldwide, they protect against only a limited number of oncogenic genotypes, and there is limited vaccine efficacy in people already infected by HPV. Clearly, there is a need to identify other modalities and alternative cost-effective means for preventing and/or treating HPV infection. With the identification of vimentin as a viral restriction factor preventing HPV16-PsV infection, approaches in drug design based on molecule structure which can eventually be incorporated into microbicides or lubricants to prevent HPV infection are conceivable (65).

## MATERIALS AND METHODS

### Cell culture.

The spontaneously immortalized human keratinocyte cell line HaCaT, the human epithelioid cervix carcinoma cell line HeLa (ATCC CCL-2), and the virus packaging cell line 293TT (established from primary embryonal human kidney cells transformed with modified human adenovirus) (66, 67) were grown and maintained in Dulbecco modified Eagle medium (DMEM; Life Technologies) supplemented with 10% heat-inactivated fetal calf serum (Biochrom), 100 U/ml of penicillin, and 100 μg/ml of streptomycin. CHO-K1 (Chinese hamster ovary K1; ATCC CCL-61) and pgsD677 (heparan sulfate-deficient cells derived from CHO-K1; ATCC CRL-2244) cells were grown and maintained in Ham's F-12 K medium (Life Technologies) supplemented with 10% heat-inactivated fetal calf serum, 100U/ml of penicillin, and 100 μg/ml of streptomycin. The spontaneously immortalized human keratinocyte cell line NIKS (ATCC CRL-12191) (58, 68, 69) was grown and maintained in F medium (3:1 [vol/vol] Ham's F-12 K medium-DMEM, 5% heat-inactivated fetal calf serum, 0.4 μg/ml of hydrocortisone [Calbiochem], 5 μg/ml of insulin [NovoRapid], 8.4 ng/ml of cholera toxin [Sigma], 10 ng/ml of recombinant human epidermal growth factor [Life Technologies], 24 μg/ml of adenine [Sigma], 100 U/ml of penicillin, and 100 μg/ml of streptomycin). All cells were grown at 37°C in a 5% CO_2_–95% air humidified atmosphere.

All cell lines have been authenticated by IDEXX BioResearch, Columbia, MO.

### HPV16 pseudovirion preparation, labeling, and quality controls.

HPV16-PsVs encapsidating the luciferase reporter gene plasmid pGL3-control (Promega) were produced in 293TT cells by cotransfection with plasmid pXULL, which encodes codon-optimized HPV16 L1 and L2, by following published procedures (67, 70). Where indicated, the virions were labeled with Alexa Fluor 488 succinimidyl ester (AF488; Life Technologies) before purification by CsCl density gradient centrifugation as described previously (30). To assess the purity of the virus stock, the purified preparations of HPV16-PsVs were subjected to SDS-PAGE and subsequent silver staining using a Pierce silver stain kit (Thermo Scientific). The protein content of the HPV16-PsV stock was quantified with a Pierce bicinchoninic acid (BCA) protein assay kit (Thermo Scientific). Furin-precleaved pseudovirions were produced after CsCl gradient centrifugation by incubation of approximately 1 μg of HPV16-PsVs with 1 U of recombinant human furin (Sigma) at 37°C for 16 to 20 h in the presence of 100 mM HEPES (pH 7.5), 0.5% Triton X-100, and 1 mM CaCl_2_. Neutralization assays were performed by incubation of the HPV16-PsVs with the neutralizing antibody H16.V5 (positive control) or H18.J4 (negative control) at a final concentration of 1:1,000 for 1 h at 4°C (70). Neutralized HPV16-PsVs were then added to the cells, and infectivity was measured as luciferase activity as described below.

### Negative-stain electron microscopy.

Both uncleaved and FPC HPV16-PsVs at a final concentration of 0.1 μg/μl were prepared for electron microscopy by washing in HSB buffer (25 mM HEPES [pH 5.7], 0.5 M NaCl, 0.02% Brij 58, 1 mM MgCl_2_, 100 μM EDTA, 0.5% ethanol) and purification through an Amicon Ultra-4 filter device (100,000-kDa molecular mass cutoff). A 3-μl drop of sample solution was then adsorbed to a glow-discharged carbon-coated copper grid (Agar Scientific) for 30 s, washed with 2 drops of deionized water, and stained with 2 drops of 2% aqueous uranyl acetate (SPI Supplies). The air-dried grid was then viewed with a Philips Tecnai F20 transmission electron microscope equipped with a field emission gun operating at 200 kV. Imaging was done with a Gatan US 4000 4kX4k charge-coupled-device (CCD) camera using the Digital Micrograph software suite.

### Pseudovirus binding and internalization assay.

For monitoring HPV16-PsV attachment and uptake, pgsD677, HeLa, HaCaT, and NIKS cells were seeded in 6-well plates at a density of 2 × 10^5^ per well and grown overnight unless otherwise specified for HeLa and NIKS cells (see “Transfection assays” below). AF488-labeled HPV16-PsVs (with or without prior furin treatment as indicated) were added to the cells at a density of approximately 4 pg/cell for 1 h at 4°C to synchronize virus attachment and to study viral binding. For virus binding assays, cells were rinsed with phosphate-buffered saline (PBS) and either prepared for confocal microscopy (see below) or lifted with 4 mg/ml of lidocaine hydrochloride (Sigma) in PBS supplemented with 10 mM EDTA. For internalization assays, cells were shifted to 37°C for 30 min unless otherwise indicated. Where indicated, virions were incubated with recombinant human vimentin protein (rhVim; Peprotech) or bovine serum albumin (BSA; Sigma) for 1 h at 4°C before addition to the cells at a final protein concentration of 20 μg/ml. Cells were rinsed with PBS and lifted with 0.025% trypsin–0.01% EDTA in PBS to remove surface-bound virions, thereby allowing detection of internalized AF488-labeled viral particles by flow cytometry. Cells were washed in fluorescence-activated cell sorter (FACS) wash solution (0.5% BSA in PBS) and fixed with 1% (vol/vol) formaldehyde (Sigma) in PBS. The detection of AF488-positive cells was performed using a FACSCalibur (Becton Dickinson) together with the software CellQuest Pro. Quadrant statistics of three independent experiments performed in triplicate were used to calculate the means ± standard errors of the means (SEM), with the Student *t* test used for determination of statistical significance compared to controls.

### Pseudovirus infection assay.

For infections which were monitored by expression of the pseudoviral luciferase reporter gene, pgsD677, HeLa, HaCaT, and NIKS cells were seeded in 12-well plates at a density of 5 × 10^4^ per well and grown overnight unless otherwise specified for HeLa and NIKS cells (see “Transfection assays” below). Furin-precleaved or uncleaved HPV16-PsVs were then added at a density of approximately 2 pg/cell at 37°C for 48 h, after which cells were washed with PBS and harvested and luciferase activity was measured using a luciferase assay system kit (Promega) with the Fluoroscan Ascent FL (Thermo Fisher Scientific) according to the manufacturer's instructions. For assays of vimentin overexpression in HeLa and NIKS cells (see “Transfection assays” below), both adherent and floating cells from the supernatant were harvested and analyzed for luciferase activity. Raw luciferase data (relative light units [RLU]) were normalized against the total protein concentration of the lysates, determined with the Pierce BCA protein assay kit (Thermo Scientific). Data are presented as percentage of infection relative to control infections, which were set as 100% or 1, respectively.

### IP and Western blot analysis.

To isolate surface proteins that interact with FPC HPV16-PsVs, pgsD677 cells were grown to 80% confluence in T175 flasks, detached using lidocaine hydrochloride–EDTA in order to maintain surface protein expression, and incubated in 2 ml of complete Ham's F-12 K medium with 1 μg of furin-precleaved HPV16-PsVs per 40 μg of total protein for 1 to 2 h at 4°C to allow particle binding but not internalization. Cells were then lysed and subjected to immunoprecipitation (IP) using the HPV16-L1-specific antibody CamVir1 (Abcam) together with the Pierce coimmunoprecipitation kit (Thermo Scientific) according to the manufacturer's instructions (Fig. 2A). Precipitated proteins were separated by SDS-PAGE, visualized by silver staining using the Pierce silver stain kit (Thermo Scientific), and compared to appropriate control IPs as indicated. Individual protein bands specific to FPC HPV16-PsV interaction were determined visually, excised from the gel, and further processed for analysis by MALDI-TOF mass spectrometry at the Centre for Proteomic & Genomic Research (CPGR; Cape Town, South Africa). Proteins were identified from the mass spectrometry data using the Matrix Science database and searching the NCBI database.

Pulldown experiments were also performed in a cell-free context using 1 μg of HPV16-PsVs (with or without furin pretreatment) per 10 μg of immobilized recombinant human vimentin protein (Peprotech) together with the Pierce coimmunoprecipitation kit (Thermo Scientific).

For Western blots of either total proteins or immunoprecipitated proteins from cell lysates, 10 μg of total protein or 10 μg of input (IN) and unbound (flowthrough [FT]) sample as well as the eluate (Elu) was subjected to SDS-PAGE and subsequent transfer to nitrocellulose membranes according to conventional protocols. When purified proteins were used in immunoprecipitation experiments, 0.1 μg of each IN and FT sample as well as the eluate was loaded onto the gels. Proteins were detected using the antibodies CamVir1 (Abcam) against the HPV16-L1 protein as well as the antivimentin antibodies H84 and V9 (Santa Cruz) as indicated.

### Flow cytometry.

Analysis of total as well as cell surface vimentin expression was performed by flow cytometry as previously described (71). Briefly, pgsD677, HeLa, HaCaT, and NIKS cells were cultured in 6-well plates at a density of 2 × 10^5^ per well and grown overnight unless otherwise specified for HeLa and NIKS cells (see “Transfection assays” below). Cells were incubated with approximately 4 pg/cell of HPV16-PsVs (with or without prior furin treatment) where indicated and lifted with 4 mg/ml of lidocaine hydrochloride (Sigma) in PBS supplemented with 10 mM EDTA and fixed with 1% (vol/vol) formaldehyde (Sigma) in PBS followed by blocking with 5% BSA (Sigma) in PBS containing 0.5% saponin (Sigma) to permeabilize cells (total vimentin expression) or not (surface vimentin expression). Rabbit antivimentin H84 antibody (Santa Cruz) together with R-phycoerythrin-conjugated donkey anti-rabbit IgG (Jackson ImmunoResearch Laboratories, Inc.) was used to detect total vimentin or surface vimentin expression relative to controls. Analysis was done on a FACSCalibur (Becton Dickinson) together with the software CellQuest Pro. Quadrant statistics of three independent experiments performed in triplicate were used to calculate the means ± standard deviations (SD), with the Student *t* test used for determination of statistical significance compared to controls.

### Immunofluorescence and confocal microscopy.

pgsD677, HeLa, HaCaT, and NIKS cells were seeded on 10-mm sterile glass coverslips at a density of 1 × 10^5^ per well in 12-well plates and grown overnight at 37°C. For cell surface labeling and colocalization experiments, 5.5 pg of AF488-labeled FPC HPV16-PsVs per cell was added and allowed to bind to cells for 1 h at 4°C, then rinsed with cold culture medium, and incubated with a goat antivimentin polyclonal antibody (Sigma; V4630; dilution of 1:100 in culture medium) for 1 h at 4°C. Cells were then rinsed with cold PBS and fixed with 4% (vol/vol) paraformaldehyde in PBS for 10 min at room temperature, washed 3 times for 5 min with PBS, and blocked with 1% BSA in PBS (blocking solution) for 1 h at room temperature before being stained with a Cy3-conjugated donkey anti-goat antibody (Jackson ImmunoResearch Laboratories, Inc.; 1:1,000 in blocking solution) for 90 min at room temperature. Cells were then counterstained with Hoechst (Sigma; 1:2,000 in PBS), washed in PBS, and mounted on glass slides with Mowiol. Slides were visualized using a Zeiss LSM 880 Airyscan confocal microscope. Colocalization analysis was performed using Zen 2012 imaging software for each optical section in z-stacks of individual cells. Colocalized pixels derived from analysis of the three-dimensional (3D) data sets were then superimposed on maximum-intensity projections of cells for display. Quantification of colocalization was performed on approximately 50 cells (derived from 2 independent experiments) for each experimental condition tested, using the built-in colocalization function in the ZEN 2012 software. Colocalization coefficients for the fluorescence channels representing Cy3-labeled vimentin and AF488-labeled FPC HPV16-PsVs, respectively, as well as overlap coefficients were determined according to the method of Manders et al. (72).

### Transfection assays.

To alter protein expression, vimentin was either knocked down or overexpressed in HeLa and NIKS cells by means of transient transfection. For knockdown experiments, 1 × 10^5^ cells per well of a 6-well plate were reverse transfected with 100 nM (unless otherwise indicated) predesigned small interfering RNA (siRNA) oligonucleotides against human vimentin (Silencer Select; Life Technologies) or 100 nM scrambled nontargeting control siRNA (Dharmacon) using Lipofectamine RNAiMax (Life Technologies) according to the manufacturer's instructions.

To overexpress vimentin, HeLa and NIKS cells at a density of 1 × 10^5^ per well of a 6-well plate were reverse transfected with 1 μg (unless otherwise indicated) of human vimentin cDNA cloned into pCMV3 (Sino Biological Inc.) using TransFectin lipid reagent (Bio-Rad) according to the manufacturer's instructions. Seventy-two hours posttransfection (for knockdown experiments) or 14 h posttransfection (for overexpression experiments), cells were analyzed for total vimentin expression by Western blotting or assessed for vimentin surface expression by flow cytometry, respectively. Moreover, transfected cells were infected with AF488-HPV16-PsVs (with or without prior furin treatment) to determine binding and infectious internalization by flow cytometry and luciferase infection assay.
